# clonevdjseq: A workflow and bioinformatics management system for sequencing, archiving, and analysis of VDJ sequences from clonal libraries

**DOI:** 10.1186/s12859-025-06107-2

**Published:** 2025-07-21

**Authors:** Keith Mitchell, Samuel Hunter, Lutz Froenicke, Karl Murray, Matthew Settles, James S. Trimmer

**Affiliations:** 1 Department of Physiology and Membrane Biology, University of California Davis School of Medicine, Davis, CA USA; 2 Bioinformatics Core, Genome Center, University of California Davis, Davis, CA USA; 3 DNA Technology Core, Genome Center, University of California Davis, Davis, CA USA

**Keywords:** Clonevdjseq, Clone, Single-cell, Monoclonal antibodies, Hybridoma, NGS, Nf-core, Nextflow, Django, Bioinformatics, TCR sequencing, BCR sequencing, VDJ, Database

## Abstract

**Background:**

Advances in next-generation sequencing technologies have facilitated extensive analysis of B cell and T cell receptor (BCR/TCR, respectively) sequences from monoclonal hybridoma libraries, single B cells, and single T cells, generating vast amounts of important data pertaining to antigen recognition. However, existing workflows and bioinformatics tools often lack the flexibility and scalability needed to handle large clonal level datasets effectively. An initial system and hybridoma dependent version of this code was distributed as part of the NeuroMabSeq publication, but clonevdjseq aims to be a technical addendum for broader system compatibility and enhanced modeling.

**Results:**

We present clonevdjseq, an integrated and accessible software solution leveraging nextflow and Django. Developed primarily for large hybridoma libraries, the workflow and pipeline is amenable to BCR/TCR sequence analysis of homogenous populations or clones of B and T cells, respectively. The clonevdjseq pipeline includes modules for read processing, amplicon denoising, and quality control of paired variable light/heavy chains of BCRs from B cells and hybridomas, or alpha(ɑ)/beta(β) and delta(δ)/gamma(γ) chains of TCRs in the case of T cell applications. The pipeline is built upon a robust, high-throughput library prep protocol, upon which processed data has been verified across thousands of monoclonal antibodies. The results of this effort has yielded sequences used to develop functional recombinant monoclonal antibodies and single chain variable fragments as a part of the NeuroMabSeq initiative where thousands of hybridoma samples were processed (Mitchell et al. in Sci Rep 13(1):16200, 2023) as well as provide additional modeling and extensibility to other modalities. The clonevdjseq software is accessible via Nextflow and also offers a database and web app as a final optional step in the processing for dissemination of results and data exploration.

**Conclusions:**

clonevdjseq offers a comprehensive and scalable solution for the processing and analysis of large monoclonal and oligoclonal VDJ datasets. Its modular design, dynamic pipeline, and robust database integration facilitate efficient data management and analysis. The platform is publicly available and aims to support the research community by providing an accessible and flexible tool for archiving and dissemination of BCR sequences from hybridomas, with applicability for other applications such as TCR sequences from single-cell T cell populations.

**Supplementary Information:**

The online version contains supplementary material available at 10.1186/s12859-025-06107-2.

## Background

The variable (V), diversity (D), and joining (J) regions of B cells and T cells undergo V(D)J recombination via rearrangement of gene segments to generate highly specific B-cell receptors (BCRs) and T cell receptors (TCRs). Antibodies (BCRs) and other varieties of antigen specific molecules are of vital importance to the biotech community. Various approaches exist for sequencing antibody sequences, each with their own advantages and disadvantages. These techniques include but are not limited to obtaining sequences for antigen-specific antibodies from hybridoma cultures, B-cell clones, as well as recent advances in single cell approaches for antigen specific B cells [2, 3]. As a result, there is an array of software applications designed for these approaches [4]. High throughput multiplex approach designed for clone based V(D)J characterization have clear advantages over Sanger and non-multiplexed Illumina or short read platforms [1, 5], yet no software package and universal workflow exists for handling such data.

Few software and workflow solutions exist for multiplexed V(D)J clone sequences on the Illumina platform. There are software packages and library preps that exist for the Nanopore platform such as NAb-seq, however read accuracy is favored on short read platforms. In addition, the short read platforms, such as Illumina, are ubiquitously available and are typically more cost efficient at larger scale compared to Nanopore instruments [6–8]. On the other hand, software applications for short-read data are typically non-clone based and are dependent on multiple workflows. For example, the well-known CellRanger software from 10 × Genomics is dependent upon Unique Molecular Identifiers (UMIs) as well as cell and sample barcodes, and as such is not applicable to the proposed workflow-dependent software [9]. Additional VDJ focused software exists on the nf-core framework such as the tool airrflow, yet they are geared towards repertoire analysis as opposed to clone based, multiplexed samples [10].

With help from the insights from large monoclonal hybridoma-based antibody sequencing initiatives, clonevdjseq effectively enhances, packages, and distributes these methodologies to support comprehensive studies of BCR/TCR sequences from monoclonal samples. This facilitates the archiving of well-characterized hybridoma and B-cell lines secreting antibodies, which is crucial for the rapid development and distribution of recombinant antibodies for research tools and therapeutics in forms such as recombinant monoclonal antibodies (R-mAbs) and single chain variable fragments (scFvs). TCR sequences may also be characterized in monoclonal or oligoclonal samples based on similar protocols with an exchange of primers from the constant regions neighboring the V(D)J regions [11].

A key strength of clonevdjseq is its applicability in contexts where single-cell sequencing approaches may be considered overly granular or cost-prohibitive or when molecules are generated from groups with preference for hybridoma and cloning approaches. This was demonstrated using a multiplexed 96-well plate approach to maintain chain pairing information [3]. While single-cell technologies provide detailed insights at an individual cell level, they often entail high costs, reagents, and equipment in addition to a complex workflow that is not always justified, especially when the target population is known to be monoclonal or highly homogeneous. Target hybridoma, T-cell, and B-cell clones can be characterized, selected for, and isolated using ELISA screens, subcloning via limiting dilution, and FACS based sorting into a single-cell well plate format [11–13]. In such cases, clonevdjseq offers a more streamlined and efficient method for capturing the full BCR/TCR diversity without the overhead costs associated with single-cell techniques.

The clonevdjseq software tool is designed to advance the study and archiving of characterized antibodies derived from monoclonal samples, significantly impacting both research and therapeutic development via peptide specific tool molecules. This software addresses the crucial need for precise and reproducible analysis of B-cell and T-cell receptors from well-studied homogenous populations.

## Implementation

### Packaging and overall design

Plates are prepared from samples of interest, and linked with paired-end read files, primer files, barcode files, and plate/well metadata which contains info on targets, sample names, and verification info. This information is processed per plate and then amplicon sequence variants (ASVs) are determined for each well, aggregated across all plates, and reported along with quality assessment reports. Finally, detailed annotation and web tools for exploration of data are made available to users. Docker containers are available for each step in the implementation.

**Fig. 1:**
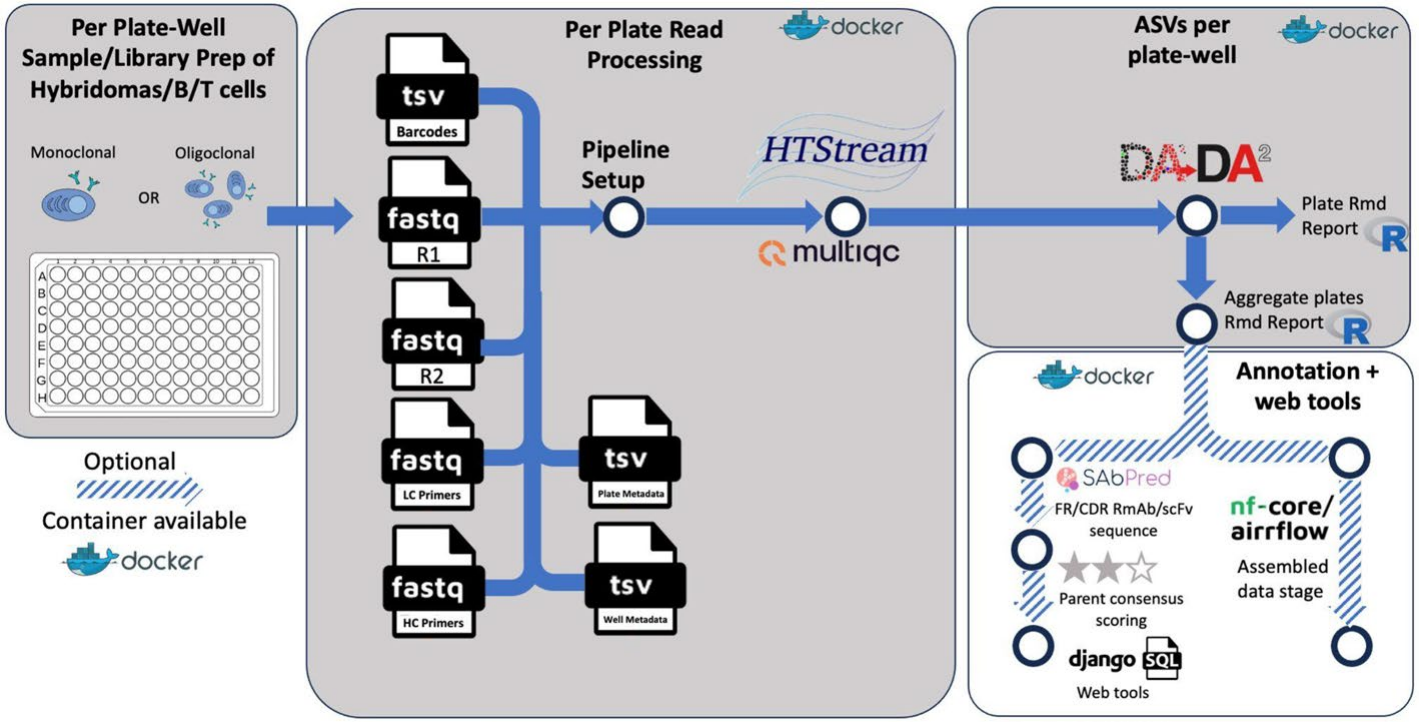
Outline of processing and analyzing VDJ sequence data from hybridomas or B/T cells

The clonevdjseq software is designed for a user-friendly deployment in diverse computational environments (Fig. 1). The software harnesses the power of Nextflow and the nf-core framework, a workflow management system that enables scalable and reproducible scientific workflows using software containers [14]. Critical components of clonevdjseq are containerized using Docker, which encapsulates specific parts of the Nextflow pipeline, ensuring that they run consistently across different computing environments. Additionally, for parts of the workflow that benefit from a more flexible setup, clonevdjseq utilizes a Conda environment, allowing for easy management of dependencies and software packages [15]. The data, formatted as FASTQ files, is then processed through multiple steps involving tools like HTStream and MultiQC for quality control with custom primer files for target constant regions of interest in samples. The workflow integrates DADA2 for identifying amplicon sequence variants and employs various tools like SAbPred and Django for annotation and web-based applications. Optionally, the data is managed and integrated using nf-core/airrflow, with an emphasis on creating further detailed reports and aggregate analyses such as lineage trees and germline analyses (Fig. 1).

### Library prep overview

**Fig. 2:**
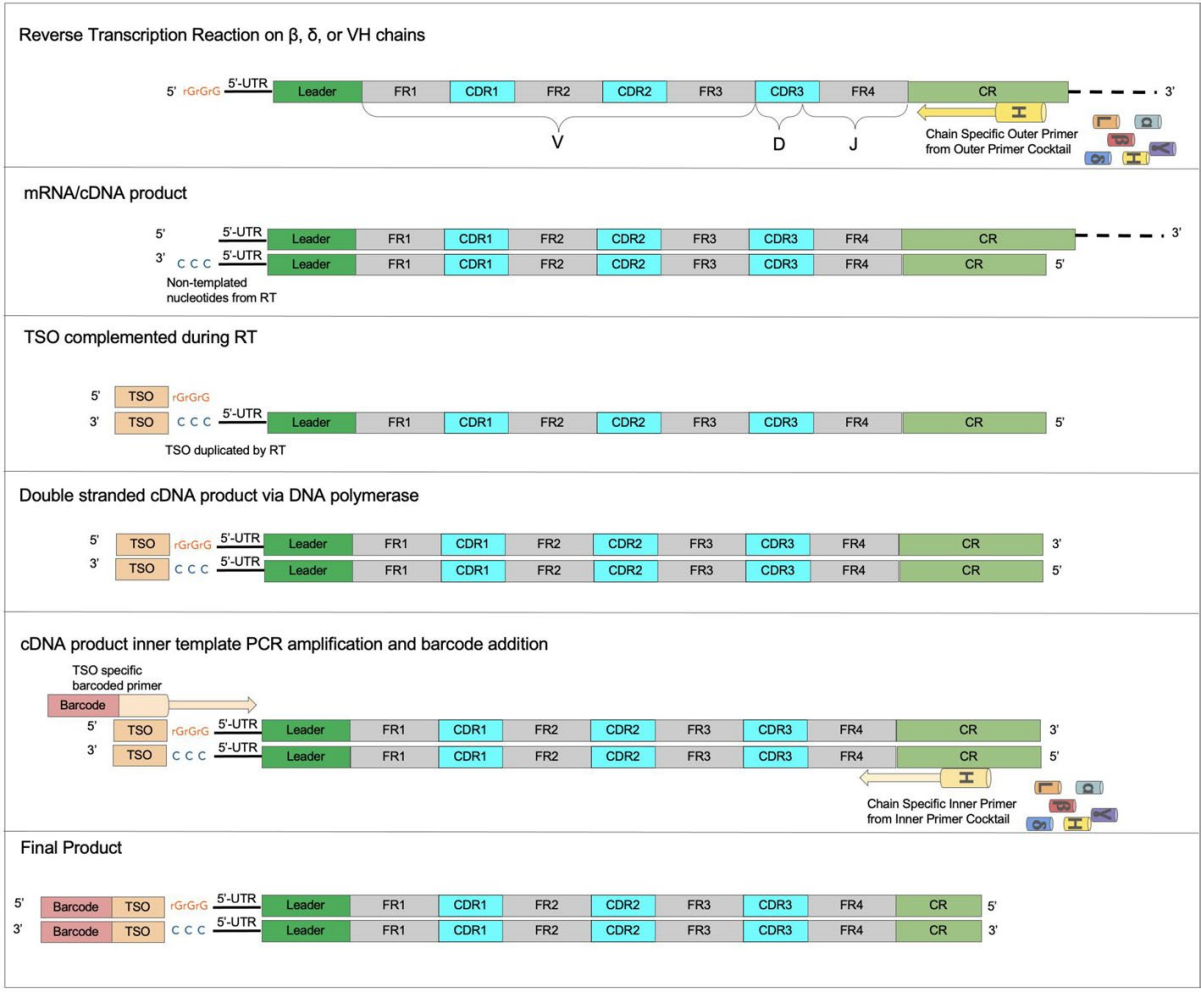
cDNA synthesis and PCR amplification overview. The schematic illustrates the positions of nested chain-specific primers used during cDNA synthesis (outer) and PCR amplification (inner). It also highlights the location of the template switching oligonucleotide. The diagram depicts the synthesis and amplification of the variable region cDNA, applicable to both variable heavy and light chain (V_H_ and V_L_, respectively) domains in BCRs, as well as Vα and Vβ domains in TCRs. Key regions such as framework regions (FR), complementarity determining regions (CDR), and constant regions (CR) are also indicated

The process begins with cDNA library preparation from either monoclonal or oligoclonal samples in the context of hybridomas or single-cell or clone isolates of B-cells and T-cells (Fig. 2). The process employs constant region primers at the 3′ end, and a template switching oligonucleotide (TSO) at the 5′ end, followed by semi-nested PCR amplification and subsequent sequencing on the Illumina MiSeq platform [1]**.** The protocol is also compatible with recent generation NextSeq 2000 and Element Biosciences AVITI sequencers. Further details and helpful visuals on the library prep, reagents, barcode sequences, and primers are extensively covered in the context of hybridomas in previous work by Mitchell et al. [1], and adaptations to include primer cocktails amenable to T cells are detailed in Fig. 2.

Our library preparation protocol shares similarities with the 5′ RACE approach, particularly in its use of constant region primers. However, it is distinct in its use of well-specific barcoding, allowing for precise tracking of sequences across different wells in multiplexed plates. Additionally, the protocol can incorporate a cocktail of primers, enhancing the flexibility to target multiple chains (e.g., BCR heavy/light chains, TCR alpha/beta/gamma/delta chains) in a single run. Combined with the use of a template-switching oligonucleotide (TSO) for enhanced amplification specificity, this method was crucial in the NeuroMabSeq effort, where thousands of monoclonal antibodies were successfully sequenced and validated. These features make our approach more versatile and scalable than traditional 5’ RACE methods, especially for large hybridoma and TCR sequencing projects.

### HTStream, denoising, and CDR/FR prediction

Both forward and reverse sequencing reads undergo a stepwise cleaning process using the HTStream package. Initially, read-pair sequences are joined and demultiplexed. To differentiate between variable light/heavy chain sequences for B-cells and hybridomas, or alpha(ɑ)/beta(β) and delta(δ)/gamma(γ) chains in the case of T-cell applications, primer sequences are identified and excised. Similarly, TSO sequences are detected and removed. Sequences containing the 'N' character, indicative of ambiguity, are also excluded from further analysis. Additionally, any low-quality base pairs with q-values below 10 are trimmed from the 3’ ends. The paired reads that overlap and meet a minimum length of 385 base pairs are then denoised and demultiplexed. These sequences are processed into amplicon sequence variants (ASVs) using the DADA2 algorithm and further processed through a specialized R and Python scripts [16]. Subsequently, the classified sequences are analyzed on the SAbPred platform via ANARCI to identify and annotate variable domain regions based on amino acid sequence and to number them based on the ImMunoGeneTics (IMGT) convention [17] (Fig. 1).

### Quality filtering on poor IMGT chain prediction and ASV support

To enhance data quality, sequences with subpar IMGT annotations for V_L_ and V_H_ amino acid predictions are excluded. Specifically, sequences with any framework region (FR) or complementary determining region (CDR) of zero length are removed. Additionally, ANARCI IMGT predictions are used to categorize sequences into bins, as certain non-V_L_ and non-V_H_ regions result in unique sequences identified by DADA2, despite having identical amino acid predictions within a monoclonal hybridoma. This discrepancy is likely due to edge trimming based on quality and ‘N’ values. These filtering and binning options are optional parameters in the Nextflow configuration. Lastly, ASV support minimums can be designated in the workflow. In our previous work on hybridomas, we used a 10% minimum for sequence reporting, and V_L_/V_H_ sequences not meeting that threshold read support were eliminated before final scoring [1].

### Database and interactive sequence exploration

Sequences are optionally organized in an SQLite database and made publicly available via a website developed with Django and hosted on Amazon Web Services (AWS). The use of Django, along with its object-relational mapper, facilitates the process of uploading, standardizing, updating, and analyzing structured data. The database offers access to nucleotide sequences of the light chain (covering IMGT amino acids 1–127) and the heavy chain (covering IMGT amino acids 1–128) variable domains. Further parameters specific to the project at hand such as species or origin, chain type, and project context, and frequently asked questions can be edited in the settings module in order to alter the web app’s context. Given ANARCI is agnostic to TCR/BCR annotation, detailed sequences for regions and fragments such as LFR1–LFR4, CDR-L1–CDR-L3, HFR1–HFR4, and CDR-H1–CDR-H3 are similarly provided via package functions as well as a web portal (Fig. 1) [17]. The BLAT (BLAST-like alignment tool) has also been integrated into the database and website, enabling users to compare sequences to others in the database. This BLAT tool provides insights into sequence similarities across different targets and facilitates various levels of quality control. The tool allows filtering for key categories of data and provides immediate access to scores and accession links for entries in the database. Additionally, users can browse the database for sequences, amino acid information, and recombinant-ready sequences, and use the "Blat Sequence" button to compare these sequences with others in the database. An AWS based Amazon Machine Image (AMI) that can readily host the Django app and database is available upon request (Figure S1) [18].

### Scoring sequences and entries

To give users confidence that the sequence pairings in the database are correct, results can be scored based on important experimental information contained in the sample naming schema. Furthermore, recent findings have shown that certain hybridomas are not as genetically simple as traditionally thought, with some studies reporting the presence of multiple unique light chains in presumably monoclonal populations [19]. Clonevdjseq incorporates mechanisms to address this phenomenon, specifically by removing light chains that appear in more than 50% of samples within a given run, likely representing the aberrant light chains present in Sp2/0 derived hybridomas [20]. Additionally, the scoring system in clonevdjseq plays a crucial role in identifying proper pairings, especially when multiple unique V_L_s are present. By gathering evidence from across samples and sequencing data, the scoring system is designed to elucidate the most likely true pairings, thereby reducing the impact of spurious or contaminating sequences. In previous work, mAb IDs of individual hybridoma cell lines follow a specific format, and scores can be assigned using the following example formats. We note that this specific format is specific to B cell hybridoma projects but could me modified for other sample sets:

$$mAbID = ProjectID/ParentID.subcloneID$$

A generalized naming scheme and grouping follows the following format:

$$sampleID = ProjectID/AntigenID.ReplicateID$$

In both situations above, hybridoma or the more general sample ID format, the data is formatted with the expectation that expected support for a given V_L_/V_H_, ɑ/β/δ/γ combination, would come from independent replicate samples from the same clonal cell or cell line. This is because projects typically are run in an organism specific manner leading to biological repeats. Similarly, antigen and parent specificity are expected to have repeat sequences obtained. BR denotes biological replicates, which we define as independent subcloned cells or cell lines, while TR refers to technical replicates which we define as independent samples of the same subclone. One would expect that independent samples from subclones of the same parental cell line to have matching light or heavy chain sequences.

Sequences may be assigned a score based on a heuristic where the ASV score ceiling (μ), with a default max score of 2 points, and the match score ceiling (λ), with a default max of 3 points, are additive to create a maximum of 5. In addition, the technical replicate weighting (ν) ranges from 0–1, where 1 treats it as significant to scoring as a biological replicate and 0 rewards no support for technical replicates.

$${\text{Total}}\,{\text{score}} = {\text{max}}\left( {\uplambda ,\ln \left( {Match\,Score} \right)} \right) + \mu \cdot ASV\,Score$$

$$ASV\,Score = \frac{Read\,Per\,Sequence}{{Read\,Per\,Primer\,Well}}$$

$$Match\,Score = \left( {BR + \left( {\nu \cdot TR} \right)} \right) - \left( {1 - \frac{BR + TR}{{Total\,Replicates}}} \right)$$

where

- $$0 \le \nu \le 1$$
- $$\mu = 5 - \lambda \left( {Default:\mu = 2} \right)$$
- $$\lambda = 5 - \mu \left( {Default:\lambda = 3} \right)$$
- *ASV Score* ∈ [0, 1]
- *Match Score* ∈ (0, ∞)
- *Total Score* ∈ (0, 5)

**Fig. 3:**
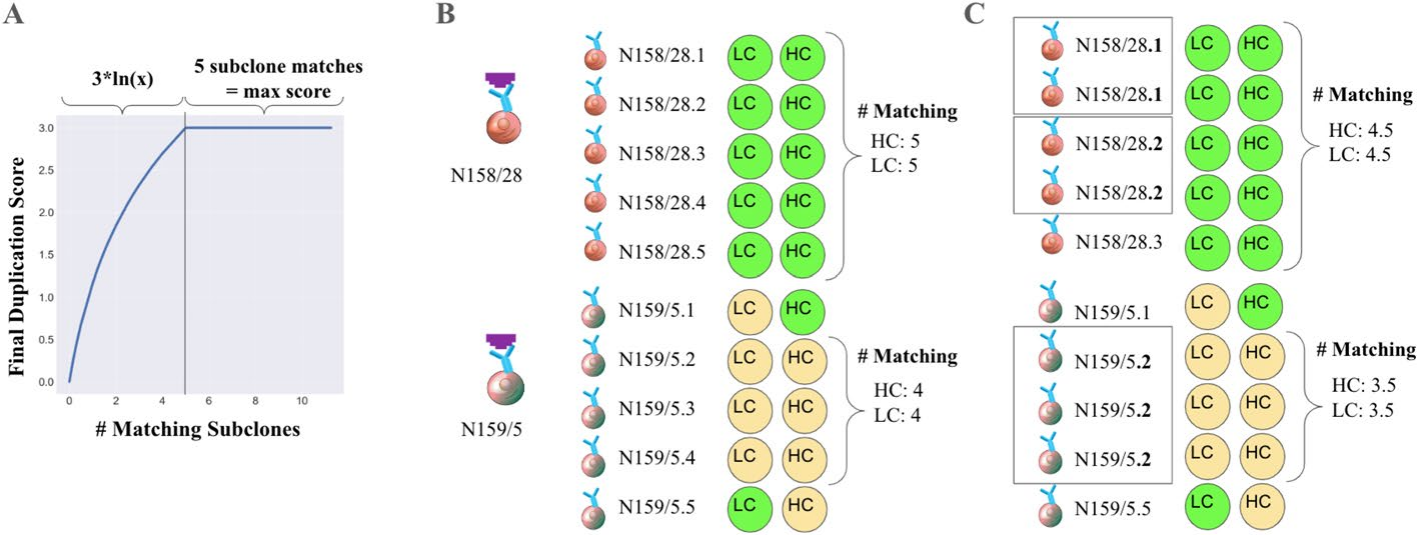
Example Overview of Heuristic Scoring. **a** Overview of the duplication score formula for a given number of matching subclones. **b** Demonstrates scoring where all sequences from independent BRs match and are unique to that cell line (samples from the hybridoma cell line expressing the N158/28 mAb), versus those that contain both matches and mismatches across BRs (samples from the hybridoma cell line expressing the N159/5 mAb), leading to increased and decreased scores, respectively. **c** Demonstrates scoring where the dataset includes some subclones that are identical (i.e., are TRs shown in boxes) and the impact of that on scoring under circumstances otherwise identical to panel A. For example, although N158/28 has 5 matches it does not receive the maximum score based on panel A. This is similarly shown for N159/5 where ¾ of the matches come from TRs so the relative final duplication score is further downgraded. These example IDs (N158/28 and N159/5) are taken from the NeuroMabSeq project with the data provided as an example to help understand the sequence support formula (1)

The scoring system, which we termed star rating, returns scores on a continuous scale ranging from 0 to 5 based on read support, defined as the ratio of a particular ASV relative to total reads produced for that sample, as well as consistency across biological and technical replicates of the same clonal line. To elucidate the true biological clonality of parents based on the many subclone sequences the following methodology is utilized. The sequences can be grouped by chain type, sequence, and features of the sample ID to count the number of occurrences indicative of TR/BR. This results in the number of sequence repeats for every entry in the database at a certain quality as well as the experimental information from which these occurred (Fig. 3).

In addition, pre-trained models and an analysis framework are provided as part of the clonevdjseq package, allowing users to build on existing work and further refine their analyses. These models, built on previously trained data from the NeuroMabSeq effort, include results where sequences were used to generate recombinant monoclonal antibodies (R-mAbs) and successfully evaluated as well as those that had the highest confidence based on the sequence support scoring system. These V_L_/V_H_ pairings were then compared against scrambled V_L_/V_H_ pairings across all results of the sequencing effort. While model accuracy is not reported as a direct indicator of success, given the variability in laboratory procedures and cell types being processed, these models provide guidance on which sequences are optimal for further experimentation but may need further refinement for the research project in question. PyIR provides additional analysis on germlines and alignments for all sequences in the database which are used in modeling of data and establishing a model to predict “High-Confidence” vs “Scrambled” pairings [21] High-confidence is established as > 4 based on the proposed scoring system for the V_L_ and V_H_, in addition to being unique sequences across pairs deemed high confidence. If multiple are > 4 the maximum is taken for the V_L_ and V_H_ and additional samples that have been verified by cloning, regardless of score, were included as well. Clonevdjseq offers both the models themselves and the data used to train them, enabling users to continue building models and defining proper pairings in oligoclonal samples. This also supports predictions of functional pairings that may lack sample-wide support needed for the match based scoring system.

## Results

### Compute performance

The total runtime for processing 10 plates was 2 h, 29 min, and 45 s, utilizing 37.5 CPU-hours. The workflow successfully completed 1931 tasks. Resource usage varied by process, with CPU usage peaking significantly due to multi-threaded processes and memory usage ranging from 1 to 4 GB per task. Key processes like runHTStream and dada2ASVs had efficient throughput, with execution times between 40 s to just over 1 min. Memory allocation was stable, maintaining physical RAM usage around 214.273 MB per task, with peaks at approximately 1.7 GB. I/O operations showed consistent read and write performance, essential for tasks involving demultiplexing and sequence trimming. For optimal performance, a setup with a multi-core processor (8 cores or more), at least 16 GB RAM (preferably 32 GB), high-speed SSD storage, and a Unix-based operating system is recommended. This configuration ensures efficient processing of monoclonal and oligoclonal hybridoma libraries using the clonevdjseq pipeline (Figure S2). The software was tested on MacOS Monterey and Ubuntu 24.04 but given the system has docker and optional conda support, system specificity should rarely be an issue.

### Web interface

**Fig. 4:**
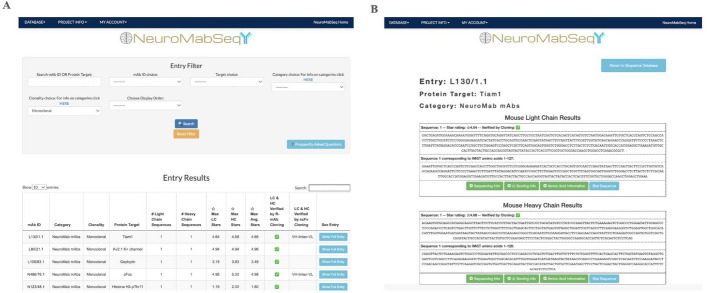
Example of website sequence entry and query capabilities. **a** Example view of the query interface that the website provides where users can search by mAb ID, target, etc. **b** The light and heavy chain entries from the sample from the L130/1.1 hybridoma subclones are shown with the different types of supporting information provided: “Sequencing Information” as well as “Scoring Information” and “Amino Acid Information”. Amino acid ANARCI annotation regions details as well as scoring and metadata information are available as further dropdown information for any given V_L_/V_H_ entry

The clonevdjseq pipeline and infrastructure was used in the NeuroMabSeq initiative and project https://neuromabseq.ucdavis.edu/ (Fig. 4). The nucleotide sequence corresponding to the ANARCI prediction of IMGT amino acids is also shown to facilitate generation of recombinant mAbs. The “Sequencing Information” dropdown contains data such as the number of ASVs attributed to the obtained sequence, the number of total reads attributed to light chains or heavy chains for the sample, as well as the plate. The “Scoring Information” reveals the star rating assigned to the sequences as well as the percentile of the rating and the portions of the scoring that constitute that scoring such as the ASV based component and replicate based component of the score. The “Amino Acid Information” dropdown contains information such as the full amino acid sequence as well as the ANARCI prediction of IMGT amino acids and regional breakdowns of FR 1–4 and CDR 1–3. In addition, the “BLAT Sequence” feature is available to compare any sequence to all other sequences in the database.

## Summary of data processed with comparison of monoclonal and oligoclonal samples

**Fig. 5:**
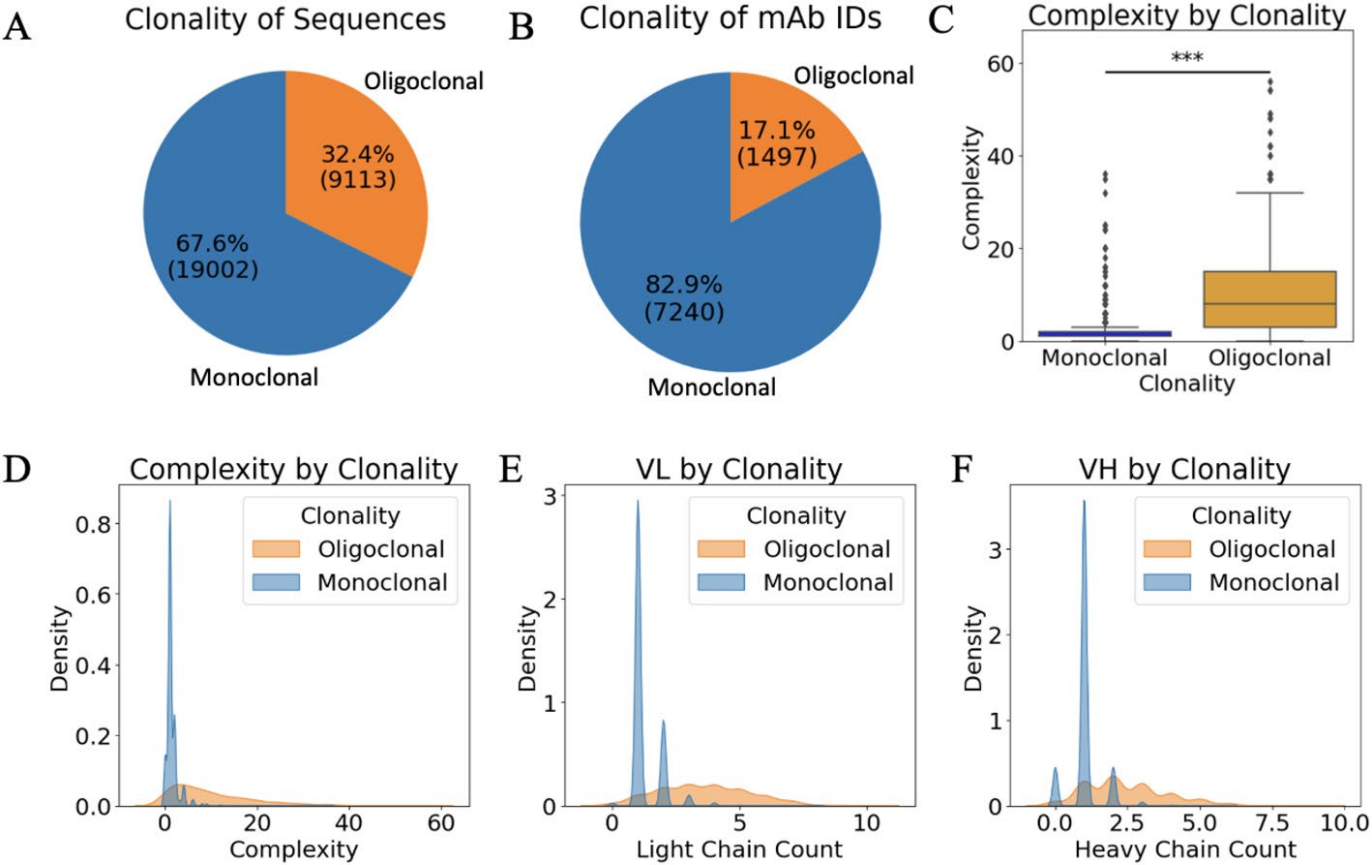
Monoclonal versus Oligoclonal comparison across the NeuroMabSeq hybridoma sequencing effort. **a** Sequence count distribution pie chart illustrates the distribution of sequence counts across different clonality groups (Monoclonal and Oligoclonal). **b** MabID count distribution displays the distribution of counts for unique MabIDs, providing insight into the prevalence of various antibodies within the dataset. **c** Complexity distribution by clonality box and whisker plot compares the complexity distribution between Monoclonal and Oligoclonal groups. The plot shows the median, quartiles, and potential outliers in complexity values for each group. A significance test (t-test) was conducted to assess differences between the two groups, with a significant *p* value indicated above the plot. **d**–**f** Kernel density estimates (KDE) illustrating the distribution of complexity, Light chain counts (V_L_), and Heavy chain counts (V_H_) across Monoclonal and Oligoclonal categories

The NeuroMabSeq project presented the opportunity to explore the diversity and interpretability of monoclonal and oligoclonal sample sequencing efforts. Unlike monoclonal antibody sequencing, where each antibody is derived from a single B cell clone/hybridoma, oligoclonal data involve multiple clones, each contributing different combinations of V_L_ and V_H_ sequences. The clonevdjseq software project experimentally included sequencing of oligoclonal samples with the hopes of determining most likely productive pairings from these samples (Fig. 5). Complexity is calculated as the total number of possible combination for a given sample (count of V_L_ * count of V_H_). This complexity, with an upper end of ~ 30, poses a significant challenge for predicting which pairings will result in functional antibodies with high specificity and affinity to the target of interest that was immunized.

### Additional model performance on NeuroMabSeq data

In addition to the provided software package, models are provided as well. Model testing was generated using the NeuroMabSeq hybridoma database where high quality pairs and scrambled, randomly paired V_L_ and V_H_ sequences are achievable from the scoring system. These high quality and scrambled pairs served as training and testing datasets and a variety of test and train ratios of the data as well as cross validation. The Gradient Boosting model based on PyIR analysis of the data has shown notable performance, with an accuracy of 93.96%, precision of 92.94%, recall of 93.96%, F1 score of 91.62%, and an ROC-AUC of 0.8097. These statistics reflect the strength of the Gradient Boosting approach in identifying high-confidence sequence pairings relative to scrambled pairings, without relying on the across-sample confidence used in the star scoring system. The precision (92.94%) indicates the proportion of true positives out of all predicted positive pairings, while the recall (93.96%) shows the model’s ability to capture all relevant true pairings. The false positive rate (FP rate) can be inferred from these metrics, calculated as 1-precision, which in this case is approximately 7%. This means that for every 100 predicted positive pairings, about 7 may be false positives. As complexity increases, particularly in oligoclonal samples, the chances of encountering more false positives also rise, as the model has more potential pairings to assess. Further exploration of these data is available in the clonevdjseq repository and is updated regularly as the NeuroMabSeq effort continues generating more data.

Based on the complexity of hybridoma samples, the accuracy of predicting proper pairings decreases as the number of possible combinations increases. This improvement highlights the benefit of the model in filtering down possible pairings, particularly as complexity increases and the risk of false positives grows. For highly complex oligoclonal hybridoma samples (with complexity up to ~ 30), around three cloning attempts may be required to verify the correct pair, whereas simpler samples with lower complexity could typically be verified with only 1–2 attempts. This model was generated using the NeuroMabSeq hybridoma database where high quality pairs and scrambled are achievable via the provided sequence scoring system, and while it demonstrates strong performance it is specific and trained for data from similarly processed mouse hybridoma samples. Different cell types or a TCR-specific sample set should consider training a new model, as a truly global model for all contexts has not yet been fully defined or validated.

## Discussion

The clonevdjseq software provides an efficient and cost-effective alternative to single-cell RNA sequencing (scRNAseq) for TCR/BCR analysis, particularly when the focus is on the collective attributes of monoclonal or oligoclonal samples rather than single-cell heterogeneity. This method addresses a critical niche by offering ease and affordability for targeted research objectives.

A key feature of clonevdjseq is its library preparation protocol, which employs constant region primers and a template switching oligonucleotide (TSO) for cDNA synthesis and PCR amplification. This process, which in our case was verified across samples from thousands of distinct monoclonal antibody-producing hybridomas, ensures high-quality sequencing data that is essential for the accurate analysis and archiving of clone-based BCR/TCR sequences.

Despite its robust framework, clonevdjseq has certain limitations that represent areas for future development. As part of the nf-core ecosystem, clonevdjseq is designed to be extensible and open to further enhancements. While the software currently relies on DADA2 for ASV identification and HTStream for sequencing processing, it lacks integration with alternative tools like Unoise or Deblur, which could enhance flexibility in ASV detection, and FastQC for additional methodology regarding sequencing processing and quality control [22–24]. These tools could be integrated into clonevdjseq in future updates, providing users with more options based on their specific requirements. The modular nature of the workflow makes it well-suited for such extensions, ensuring that the platform can evolve as new tools and approaches become available.

With regards to the comparability to other tools in the space of TCR and BCR sequencing and analysis, clonevdjseq distinguishes itself through its focus on multiplexed, clonal-level, well-based analysis, which many existing platforms do not prioritize. Tools such as CellRanger from 10 × Genomics are primarily designed for single-cell sequencing, utilizing unique molecular identifiers (UMIs) and cell barcoding for tracking individual cells. While these methods provide high-resolution insights into immune repertoires, they are not optimized for clonal populations or multiplexed library preparations. On the other hand, software like nf-core/airrflow are well-suited for repertoire analysis but are limited in their ability to handle multiplexed plate-based data with well-specific barcoding, as used in clonevdjseq. This creates shortcomings with regards to retrieving functional sequence pairing, such as V_L_ and V_H_. These tools typically focus on repertoire diversity at a population level rather than the in-depth characterization of clonal BCR or TCR sequences. Clonevdjseq, by contrast, is specifically designed for generating high-quality consensus sequences from clonal libraries, making it highly suited for monoclonal antibody development and target-based research, where precision and scalability are crucial. Further distinguishing clonevdjseq is its integration with the nf-core framework, allowing for greater flexibility and extensibility compared to more rigid workflows.

While clonevdjseq fills an important niche in BCR and TCR sequencing, it is not without limitations. Its current focus is primarily on BCR data, with TCR data handling being an area of active development. Tools such as pairSEQ show promise with integration into a platform such as clonevdjseq with regards to handling of TCR data and finding proper pairings in samples with higher complexity [25]. Additionally, challenges like well contamination and clonal population size remain, though they are mitigated through the use of ASV denoising and a heuristic scoring system.

Data availability is crucial for generating R-mAbs for research antibody production, as well as for advancing antibody modeling and machine learning. Large, homogeneous datasets provide a framework for improving computational methods such as docking, epitope prediction, and in silico antibody design [26, 27]. The clonevdjseq software enhances data accessibility by providing comprehensive BCR/TCR datasets, including sequences and target amino acids. These resources are vital for refining bioinformatics tools and validating computational docking methods, especially in complex samples in which identifying the correct target peptide is challenging.

## Conclusion

Clonevdjseq fills a significant gap in the field by offering a scalable, modular, and integrated solution for processing and analyzing large VDJ datasets. Existing workflows and bioinformatics tools often lack the flexibility and scalability needed to handle such data effectively. clonevdjseq’s dynamic pipeline generation and robust database integration facilitate efficient data management and analysis, making it a valuable tool for the research community.

The multiplexing approach and software developed here could significantly impact other research areas, such as enhancing throughput in microbiome studies through targeted amplicon sequencing of rRNA subtypes using primer cocktails. It could also advance biomarker evaluation by enabling targeted monitoring with specific primer cocktails, alongside numerous other potentially groundbreaking applications in high-throughput targeted sequencing. This software and multiplexing schematic highlight the use of nested chain-specific primers for cDNA synthesis (outer) and PCR amplification (inner), as well as the role of the template switching oligonucleotide.

In summary, clonevdjseq advances the study and archiving of characterized antibodies derived from monoclonal samples, significantly impacting research and therapeutic development through peptide-specific tool molecules. It fulfills the crucial need for precise and reproducible analysis of B-cell and T-cell receptors from well-studied homogeneous populations.

## Availability and requirements

Project name: clonevdjseq.

Project home page: https://github.com/keithgmitchell/nf-core-clonevdjseq.

Operating system(s): Platform independent.

Programming language: Nextflow, Bash, Python, R.

Other requirements: Docker or Conda.

License: MIT License: Free use with attribution, no warranty, no liability.

Any restrictions to use by non-academics: No restrictions mentioned for non-academic use.

## Supplementary Information

Additional file 1: Figure S1. Architecture of available AWS machine image. Local testing servers and data exploration tools use the django framework whereas a properly hosted web server will utilize proper parallelization and security implementation of Gunicorn and NGINX. Figure S2. The clonevdjseq pipeline was executed on a Mac with a 2.3 GHz 8-Core Intel Core i9 processor, 16 GB 2400 MHz DDR4 memory, and Intel UHD Graphics 630 1536 MB. These are the results of running 10 plates with full sequencing results in parallel with Nextflow. Typically, each of the paired end read files range from 100MB to 500MB.

## Data Availability

No datasets were generated or analysed during the current study.
