# Supplementary material for: clonevdjseq: A workflow and bioinformatics management system for sequencing, archiving, and analysis of VDJ sequences from clonal libraries
Source: BMC Bioinformatics. 2025 Jul 21;26:186. doi: 10.1186/s12859-025-06107-2 (PMC12278597; doi:10.1186/s12859-025-06107-2)
Supplement: Supplementary file 1 — Additional file 1: Figure S1. Architecture of available AWS machine image. Local testing servers and data exploration tools use the django framework whereas a properly hosted web server will utilize proper parallelization and security implementation of Gunicorn and NGINX. Figure S2. The clonevdjseq pipeline was executed on a Mac with a 2.3 GHz 8-Core Intel Core i9 processor, 16 GB 2400 MHz DDR4 memory, and Intel UHD Graphics 630 1536 MB. These are the results of running 10 plates with full sequencing results in parallel with Nextflow. Typically, each of the paired end read files range from 100MB to 500MB. [file 12859_2025_6107_MOESM1_ESM.docx]

# **Extra Materials**

#
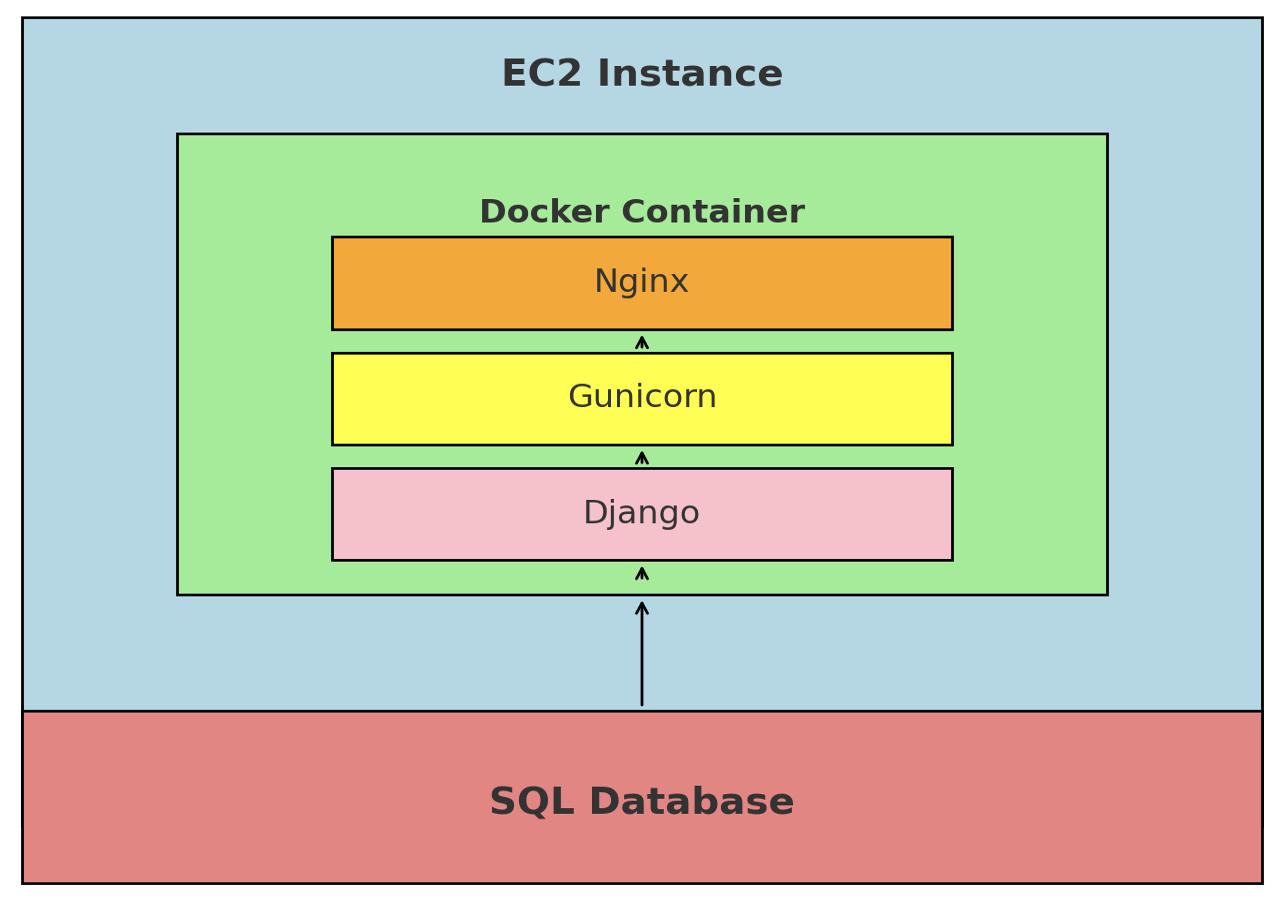


**Figure S1:** Architecture of available AWS machine image. Local testing servers and data exploration tools use the django framework whereas a properly hosted web server will utilize proper parallelization and security implementation of Gunicorn and NGINX.

# **
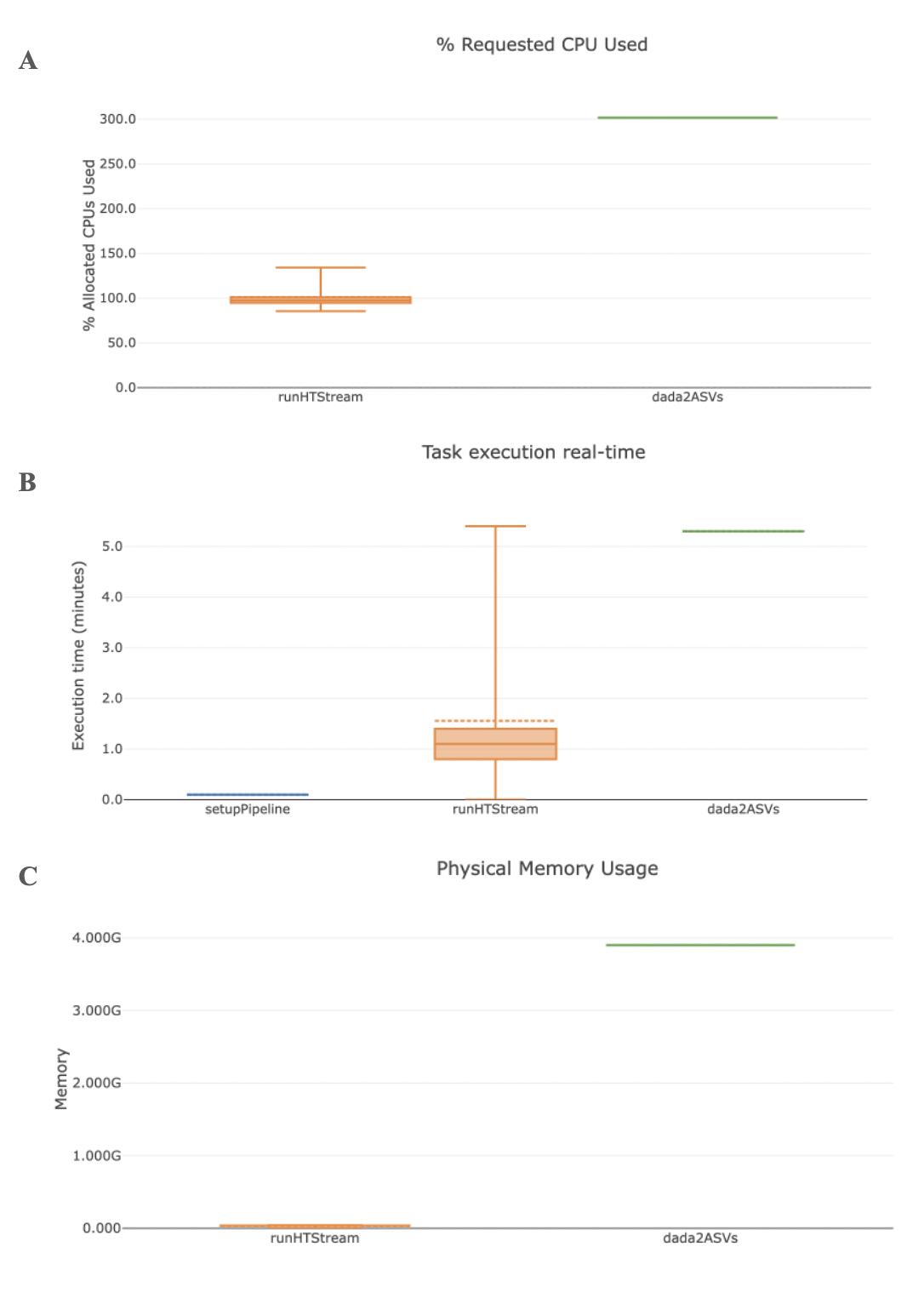
**

**Figure S2:** The clonevdjseq pipeline was executed on a Mac with a 2.3 GHz 8-Core Intel Core i9 processor, 16 GB 2400 MHz DDR4 memory, and Intel UHD Graphics 630 1536 MB. These are the results of running 10 plates with full sequencing results in parallel with Nextflow. Typically, each of the paired end read files range from 100MB to 500MBs.
